# On the Discovery of Aromatic Vinegar

**Published:** 1803-04-01

**Authors:** William Henry

**Affiliations:** Manchester


### *To the Editors of the Medical and Physical Journal.*

GENTLEMEN,

I Am sensible that an apology is necessary for obtruding on you and on your readers, a subject which may appear, at first view, to have little claim to general attention. Were the rights and interests of an individual alone involved on this occasion, I should not have requested a place in your Journal for the following statement. But it is surely a matter of general concern, that the appropriation of inventions and improvements should be dealt with strict justice to their authors. For the prospect of this distribution of "honour where it is due," is one of the most animating principles of action; and the extinction of this motive would follow an indifference, on the part of the public, to the claims of inventors.

More than fifteen years ago, during the delivery of a course of chemical lectures in this town, by my father, he had occasion to notice a quality of acetic acid, or radical vinegar, which had not, to his knowledge, been before observed, viz. its property of dissolving camphire, and various essential oils. The compound was found to possess a most agreeable and pungent odour; and as the *vinaigre des quatres voleurs* had gained much reputation in preventing infection, it occurred to him that the newly discovered solution would have still more powerful effects, in consequence of its high state of concentration. A bottle of this preparation he gave to a late active magistrate and philanthropist, (T. B. Bayley, Esq. F. R. S.) who, in the course of an unwearied and undaunted exercise of his public functions, was frequently exposed to the dangers of foul and infected air. Mr. Bayley was highly gratified with its effects; and not only made constant use of the aromatic vinegar on the bench, and on his visits to the prison, but introduced it to the adoption of several of the judges, and principal gentlemen at the bar. He, also, first suggested to my father, the propriety of benefiting by his discovery, and and was the medium of a connection with Mr. Bayley, perfumer, in Cockspur Street, London, which has continued to the present day.

The aromatic vinegar, like every other article in general demand, has been a frequent subject of imitation. But it is not of this that I complain; for in consequence of unremitting assiduity, our preparation has maintained a superiority, both in quality and extent of sale, over all others. The occasion of this appeal to your readers is, that one of these imitations has been lately sanctioned by the name of a respectable physician, who, though not expressly, yet by implication, has bestowed on another the credit of that invention, which, in justice, is due to my father. (See an advertisement in the public papers from a druggist in London, containing a letter from Dr. Trotter, Physician to his Majesty's Fleet).

From the recommendatory letter of Dr. Trotter, it is evident that he was ignorant of any prior claim; and he was, therefore, made acquainted by my father, in the most respectful manner, with the facts which have been already laid before you. To this letter the Doctor has made no reply, though he declared verbally, to a medical gentleman, that my father's preparation had never happened to fall in his way; but that, if it had, he should with equal readiness have given testimony in its favour. The advertisement, however, continues to be regularly inserted; and I therefore deem it expedient thus publicly to appeal against the injustice of such a proceeding; especially in favour of a man who has imitated the original only in copying, with unblushing effrontery, the words of an advertisement drawn up by myself.

I believe there are few of your readers who will not decide, that the ordinary forms of civility required Dr. Trotter to have taken some further notice of the letter which was addressed to him; that such an attention ought to have been paid to one of the oldest practitioners of medicine in this country; and that more respect was due to a man (whom I trust it is not unbecoming in me to characterise, in terms already publicly applied to him, *viris laudatis*)* "respectable in science and in literature," and "distinguished by ingenuity, honour, and the strictest integrity."

I am, &c.

*Manchester, March* 13, 1803. WILLIAM HENRY.

---

* Dr. Aikin and Dr. Percival.

